# Calling for Careful Designs for the Evaluation of Interactive Behavioral Measures on Early False-Belief Reasoning

**DOI:** 10.3389/fpsyg.2017.01302

**Published:** 2017-08-02

**Authors:** David Buttelmann

**Affiliations:** Department of Developmental Psychology, University of Bern Bern, Switzerland

**Keywords:** theory of mind, infancy research, implicit, interactive tasks, false belief

With the introduction of the interactive false-belief paradigm, Buttelmann et al. (2009) proposed that already infants track another person's beliefs in order to infer her or his goal and help accordingly. This view has been challenged by Allen (2015) who argued that is not the experimenter's mental states but the social situation the test was embedded in that influenced participants' performance.

In Buttelmann et al.'s study, 16- and 18-month-old infants and 30-month-old toddlers watched as an experimenter first opened and closed two empty boxes and then placed a toy in one of them. He then either left (false-belief condition) or stayed and watched (true-belief condition) as an assistant took the toy out of this box and placed it into the other box. The assistant then locked both boxes, out of the experimenter's sight (keeping him ignorant about the locking mechanism in both conditions). At test, the experimenter tried to open the box he had placed the toy in originally. Subsequently, the participants, who were knowledgeable about the locking mechanism, were allowed to help the experimenter by opening one of the two boxes (or touching the locking mechanism, at least). Buttelmann et al.'s rationale was that in the false-belief condition, participants might reason that since the experimenter believed this box to contain his toy, he probably wanted his toy. The appropriate help would thus be opening the other box (i.e., the one containing the toy). In the true-belief condition, in contrast, since the experimenter knew this box to be empty (and his toy being located in the other box), it was unlikely that he wanted the toy, so he seemingly wanted to open this empty box. The appropriate help in this condition would thus be opening the empty box. The authors found that children at all three ages chose the box with the toy significantly more often in the false-belief than the true-belief condition. Separate analyses of the false-belief condition further revealed that they chose this box significantly at above chance level.

Allen's (2015) alternative explanation of the findings rests on the fact that in the false-belief condition, the experimenter's “false-belief was conflated with the playing of a trick” (p. 63). That is, the assistant performed the switch of locations in a sneaky manner in the false-belief condition, whereas no such deceptive element was present in the true-belief condition. In order to test this alternative explanation, Allen first successfully replicated the result of the false-belief condition with 3- to 5-year-old children and then ran two control conditions, the clairvoyance condition and the hands-full condition. The *clairvoyance condition* was designed to test whether participants indeed applied a mentalistic understanding in the false-belief condition. The procedure in this condition was similar to that of the false-belief condition, with the crucial difference that at test the experimenter tried to open the box that actually contained the object (i.e., the one she believed to be empty). Allen's rationale was that if children interpreted the experimenter's action mentalistically, they should reason that, since she believed this box to be empty, she wanted “an empty box” (p. 66, p. 69). The appropriate way to help might thus be to open the empty box (i.e., the one the experimenter originally had put her object in). If, in contrast, the children opened the box with the object, this might suggest that the children were not basing their helping response on the experimenter's false belief. The children's performance in this condition did not differ from that in the false-belief condition: they opened the box with the toy. The *hands-full condition* was designed to directly investigate the influence of the type of social situation on participants' response. First, children were given a possible reason for why the experimenter might want an empty box: they were told that she wanted to store away toys (to hide them from a mean agent). The experimenter filled the first box with toys and left. In her absence, the assistant moved all the toys from this box to the other box. However, the replacement of the toys was not performed in a deceptive context. The experimenter then returned with some toys and tried to open the box she believed to be empty. Allen predicted that if children's response was driven by the broader social situation (i.e., the storing-away context in this condition), children might open the empty box. Results revealed that children as a group chose randomly between the two boxes. This chance-level performance resulted from 3-year-olds choosing the box with the toys, 4-year-olds choosing randomly, and 5-year-olds choosing the empty box. The author concluded that children's performance in the current study and in Buttelmann et al.'s study was based on the social situation rather than the experimenter's beliefs. In this article I attempt to clarify why I think the interpretation of Allen's results as challenging Buttelmann et al.'s findings might be invalid.

Although, there is evidence that the type of social situation a false-belief task is embedded in does influence preschoolers' performance (for playful vs. predator/prey scenario see Keenan and Ellis, 2003; for deceptive context see Wellman et al., 2001), there is no evidence for such an effect in 2- or even 1-year-olds. In contrast, at the time Allen's study was submitted there were already two studies published showing that 18-month-olds pass interactive false-belief tests even if the task was not embedded in a deceptive context (Knudsen and Liszkowski, 2012; Buttelmann et al., 2014; see also Buttelmann et al., 2015, for a more recent one). Although, this does not rule out the idea that the deceptive context in the Buttelmann et al. (2009) study boosted participants' performance, the hypothesis that such a context is a necessary precondition for infants to pass an interactive false-belief test has already been falsified.

Important theoretical and methodological differences between conditions within Allen's (2015) study make the comparison of findings from these conditions difficult. For example, while in the false-belief condition, the experimenter tried to open the empty box, in the clairvoyance condition she tried to open the box with the object (note that in Buttelmann et al.'s study the experimenter tried to open the empty box in both conditions). Thus, while in the false-belief condition children needed to decide how to weigh the experimenter's pulling cue on one box and the object's presence in the other box, in the clairvoyance condition both of these cues were located at the same box. In order to choose the empty box, participants needed to inhibit both of these cues. This might have been too demanding. Further, in contrast to the false-belief condition, in the clairvoyance condition the experimenter behaved inconsistently to her beliefs: although she believed the toy to be in the first box, she tried to open the other one. Such belief-inconsistent behavior seems to be quite challenging for children to understand and is the basis for the violation-of-expectation paradigm (e.g., Onishi and Baillargeon, 2005). There, children tend to look longer at a scene in which an agent approaches a container she believes to be empty (but does contain the object) compared to a scene in which the agent approaches an empty box she believes to contain the object (see Baillargeon et al., 2010, for a review). Following this logic, children might have been puzzled by the experimenter's belief-inconsistent behavior in this condition. Consequently, opening the box that combined both cues of the situation (i.e., the experimenter's pulling action and the object being inside this box) might be the safest option for participants, which is mirrored by the results.

Important theoretical and methodological differences between Allen's (2015) and Buttelmann et al.'s (2009) studies make the use of Allen's findings to interpret that of Buttelmann et al. difficult. Firstly, Allen's and Buttelmann et al.'s studies differ dramatically in the age groups tested (Allen: 3- to 5-year-olds; Buttelmann et al.: 16-, 18-, and 30-month-olds). In my opinion, it seems absolutely possible that results for older and younger children differ due to differences in motivation, mobility, and experience. What might happen in older children is, for example, that they take the assistant's perspective (instead of the experimenter's) and keep on playing a trick on the experimenter or they do not want to play a trick on the experimenter (both participant behaviors reported by Allen). Even though Allen replicated the false-belief condition with 3- to 5-year-olds (by using slightly different coding criteria than Buttelmann et al.), it seems impossible to conclude that infants and toddlers would perform as did preschoolers in the clairvoyance and hands-full conditions. Secondly, Allen's assumption that both the clairvoyance condition and Buttelmann et al.'s true-belief condition might be comparable is difficult given the different rationales behind those conditions. In Buttelmann and colleagues' true-belief condition, the experimenter's most likely goal is opening the specific box he tried to open. In Allen's clairvoyance condition, the experimenter's most likely goal is “an empty box” (whatever box this might be, p. 66). These two goals differ in their level of abstractness. It seems that children do not draw the inference of “wanting an empty box” before the age of 5, as demonstrated by the results in the hands-full condition in Allen's study. Thirdly, Allen's hands-full condition - designed as a control condition for Buttelmann et al.'s false-belief condition - presented participants with a completely different type of false-belief task: Buttelmann et al.'s task is an *approach* false-belief task based on the original explicit change-of-location task (Wimmer and Perner, 1983). That is, in the false-belief condition the experimenter is looking for an object to have. The logic of the hands-full condition follows that of an *avoidance* false-belief task (Leslie et al., 2004). That is, the protagonist desires to avoid the box with the toys (in order to find an empty box for her toys). Children pass explicit avoidance false-belief tasks at a significantly later age than explicit approach false-belief tasks (e.g., see Cassidy, 1998; Leslie and Polizzi, 1998). The finding that only the 5-year-olds succeeded in the hands-full condition demonstrates that the demands in this condition were extremely high. Fourthly, whereas in Buttelmann et al.'s study and Allen's false-belief and clairvoyance conditions children needed to infer the experimenter's goal, they were explicitly told the experimenter's goal in the hands-full condition. Thus, it seems thinkable that even without watching the whole procedure children could have passed this condition. The finding that only the 5-year-olds did suggests that it was difficult for (younger) children to understand this goal or the physical circumstances included in this condition.

Overall, the replication of Buttelmann et al.'s results in the false-belief condition with an older age group is interesting. However, Allen's (2015) aim was to investigate whether or not the social situation Buttelmann et al.'s (2009) task was embedded in had an influence on participants' performance. In my opinion, Allen's design does not allow to effect this purpose. I think the clairvoyance condition reveals preschoolers' difficulty to inhibit choosing the box that was the only one being enhanced at test (i.e., the one containing the object *and* being pulled) or that they have problems generalizing goals (i.e., the experimenter wants “*an* empty box”). The hands-full condition tells us something about children's difficulty and increasing ability to adjust their helping behavior to a person's observable need or explicit goal. Given the reasons outlined above I conclude that these conditions are not valid control conditions challenging the idea that infants and toddlers applied belief reasoning in the original study. A promising attempt to test the effect of the deceptive context on infants' performance in an interactive false-belief task might be to run two almost identical conditions—one including a deceptive element and one without such element—with infants from the original age group. Such a control condition in combination with others might allow valid conclusions about the degree to which behavioral measures help transcend the infant false-belief debate.

## Author contributions

The author confirms being the sole contributor of this work and approved it for publication.

### Conflict of interest statement

The author declares that the research was conducted in the absence of any commercial or financial relationships that could be construed as a potential conflict of interest.

## References

[B1] Allen J. W. (2015). How to help: can more active behavioral measures help transcend the infant false-belief debate? New Ideas Psychol. 39, 63–72. 10.1016/j.newideapsych.2015.07.008

[B2] Baillargeon R. Scott R. M. He Z. (2010). False-belief understanding in infants. Trends Cogn. Sci. 14, 110–118. 10.1016/j.tics.2009.12.006 20106714 PMC2930901

[B3] Buttelmann D. Carpenter M. Tomasello M. (2009). Eighteen-month-old infants show false belief understanding in an active helping paradigm. Cognition 112, 337–342. 10.1016/j.cognition.2009.05.006 19524885

[B4] Buttelmann D. Over H. Carpenter M. Tomasello M. (2014). Eighteen-month-olds understand false beliefs in an unexpected-contents task. J. Exp. Child Psychol. 119, 120–126. 10.1016/j.jecp.2013.10.002 24267393

[B5] Buttelmann F. Suhrke J. Buttelmann D. (2015). What you believe is what you get: infants simultaneously represent two identities of one object in a Theory of Mind task. J. Exp. Child Psychol. 131, 94–103. 10.1016/j.jecp.2014.11.009 25544393

[B6] Cassidy K. W. (1998). Three-and four-year-old children's ability to use desire-and belief-based reasoning. Cognition 66, B1–B11. 10.1016/S0010-0277(98)00008-0 9675983

[B7] Keenan T. Ellis B. J. (2003). Children's performance on a false-belief task is impaired by activation of an evolutionarily-canalized response system. J. Exp. Child Psychol. 85, 236–256. 10.1016/S0022-0965(03)00072-9 12810037

[B8] Knudsen B. Liszkowski U. (2012). 18-month-olds predict specific action mistakes through attribution of false belief, not ignorance, and intervene accordingly. Infancy 17, 672–691. 10.1111/j.1532-7078.2011.00105.x 32693489

[B9] Leslie A. M. Friedman O. German T. P. (2004). Core mechanisms in ‘theory of mind’. Trends Cogn. Sci. 8, 528–533. 10.1016/j.tics.2004.10.001 15556021

[B10] Leslie A. M. Polizzi P. (1998). Inhibitory processing in the false belief task: two conjectures. Dev. Sci. 1, 247–253. 10.1111/1467-7687.00038

[B11] Onishi K. H. Baillargeon R. (2005). Do 15-month-old infants understand false beliefs? Science 308, 255–258. 10.1126/science.1107621 15821091 PMC3357322

[B12] Wellman H. M. Cross D. Watson J. (2001). Meta-analysis of theory-of-mind development: the truth about false belief. Child Dev. 72, 655–684. 10.1111/1467-8624.00304 11405571

[B13] Wimmer H. Perner J. (1983). Beliefs about beliefs: representation and constraining function of wrong beliefs in young children's understanding of deception. Cognition 13, 103–128. 10.1016/0010-0277(83)90004-5 6681741

